# Exposure-response association between outdoor activity time and myopia risk in Chinese children and adolescents: a systematic review and meta-analysis

**DOI:** 10.7189/jogh.16.04122

**Published:** 2026-04-03

**Authors:** Jin Dai, Jing Wang, Yong-Hong Bao, Yang Zhang

**Affiliations:** 1 College of Physical Education, Hunan Normal University, Changsha, China; 2 Independent researcher, Windermere, Florida, USA

## Abstract

**Background:**

The prevalence of myopia among Chinese children and adolescents has increased sharply over recent decades, raising concern about modifiable environmental risk factors. Although outdoor activity is widely recognised as protective, the quantitative exposure-response relationship within the Chinese educational context remains incompletely defined.

**Methods:**

A systematic review and meta-analysis were conducted following PRISMA guidelines. Studies examining the association between daily outdoor activity time and myopia risk among Chinese youth were identified from major English and Chinese databases (Web of Science, PubMed, CNKI, and Wanfang) through October 2025. Random-effects meta-analysis was conducted to pool odds ratios (ORs). Prespecified subgroup and sensitivity analyses examined exposure thresholds, study design, and geographic latitude.

**Results:**

We included 31 studies comprising 380 215 participants. Greater outdoor activity was consistently associated with lower myopia risk (pooled OR = 0.75; 95% confidence interval (CI) = 0.71–0.80), although substantial heterogeneity was observed (*I*^2^ = 94%). Compared with <1 hour per day, outdoor exposure of 1–2 hours (OR = 0.85; 95% CI = 0.79–0.92), 2–3 hours (OR = 0.86; 95% CI = 0.78–0.95), and > 3 hours (OR = 0.74; 95% CI = 0.63–0.87) was associated with reduced risk. When dichotomised at two hours, exposure >2 hours yielded an OR of 0.74 (95% CI = 0.69–0.80). Findings were directionally consistent across study designs and latitude strata.

**Conclusions:**

Greater outdoor activity time is associated with lower myopia risk among Chinese children and adolescents. An approximate daily exposure of around two hours appears to represent a pragmatic benchmark under current educational conditions.

The prevalence of myopia among Chinese children and adolescents has risen sharply over the past two decades. Data from the National Survey on Student Physical Fitness and Health indicate that the proportion of school-aged youth with poor visual acuity rose by approximately 22% between 1995 and 2014. This escalation paralleled rapid expansion of higher-education enrolment – from roughly one million college admissions in 1999 to more than seven million by 2014 – and intensified academic competition that lengthened study hours, increased near-work activity, and curtailed outdoor play. Combined with rapid urbanisation and widespread digital-device use, these changes have produced an environment in which students spend most waking hours indoors under artificial light. As a result, China has experienced one of the steepest global increases in youth myopia and high myopia [1], consistent with projections suggesting that nearly half of the global population may be myopic by 2050 [2].

This rapid rise cannot be explained by genetic susceptibility alone and is largely attributed to modifiable behavioural and environmental factors [3]. Among these, reduced outdoor activity has consistently emerged as a key risk factor. Outdoor light intensity – typically 10 000 to 100 000 lux compared with less than 500 lux indoors [4] – influences ocular growth through multiple pathways. Experimental studies demonstrate that bright light stimulates retinal dopamine release, which acts as a regulatory signal inhibiting excessive axial elongation [5–8]. Natural daylight’s broad spectral composition may further contribute to ocular growth regulation [9]. Together, these mechanisms provide biological plausibility for the protective role of outdoor exposure.

Although the association between outdoor exposure and myopia risk is well established, the quantitative exposure-response relationship remains incompletely defined, particularly within China’s distinctive educational and environmental context. Previous global meta-analyses have reported modest risk reductions associated with additional outdoor time [10–12]; however, these syntheses combined heterogeneous populations and outcome definitions, limiting direct applicability to Chinese youth. Few studies have examined discrete daily exposure thresholds – such as one, two, or three hours per day – within this population.

Chinese students experience prolonged classroom instruction and homework demands that often exceed 10 hours per day, with limited recess and restricted access to outdoor spaces, especially in densely populated urban areas [13,14]. These structural factors may influence both achievable exposure levels and protective thresholds. Given these contextual differences, China provides a critical setting for examining how the amount of daily outdoor time relates to myopia risk.

Accordingly, this meta-analysis aimed to clarify the exposure-response relationship between daily outdoor activity time and myopia risk among Chinese children and adolescents, and to characterise the exposure range most consistently associated with reduced myopia incidence. Establishing this relationship provides quantitative evidence to inform school scheduling, environmental design, and public-health strategies for myopia prevention.

## METHODS

### Search strategy

This systematic review follows the PRISMA guideline. A literature search was conducted across Web of Science Core Collection, PubMed, China National Knowledge Infrastructure, and Wanfang Data to identify studies examining the association between outdoor activity time and myopia among Chinese youth. The search covered all records from database inception through October 2025.

Both English and Chinese search terms were used to ensure inclusion of domestic and international publications. For English-language databases, the following Boolean strategy was applied: ‘myopia’ OR ‘refractive error’ AND ‘outdoor’ AND ‘children’ OR ‘adolescents’ OR ‘students’ AND ‘Chinese’. Equivalent translated terms were applied in Chinese databases to capture relevant literature on myopia, outdoor activity, and school-aged populations. Reference lists of relevant reviews and eligible studies were also manually screened to identify additional articles.

### Eligibility criteria

Studies were eligible if they quantitatively assessed the association between outdoor activity time and myopia among Chinese youth. The target population primarily included participants younger than 18 years, although studies involving adolescents under 20 years were accepted if the majority were within the school-aged range.

Studies were excluded if they were reviews, conference abstracts, or reports lacking quantitative risk estimates. Studies involving Chinese ethnicity residing outside mainland China (*e.g*. Taiwan, Hong Kong, Singapore, and Australia) were excluded because differences in educational systems and environmental exposures could limit comparability with mainland populations.

### Outcome measures

The primary outcome was myopia, defined according to the national clinical standard in China as a spherical equivalent refractive error of less than −0.50 diopters. Effect measures were extracted as ORs, hazard ratios (HRs), or relative risks, where reported. One cohort study reported HRs, which were included in the pooled analysis. Because the annual incidence of myopia among school-aged children is relatively low (typically < 10% per year), ORs and HRs approximate relative risk estimates under rare-outcome conditions. Therefore, these measures were synthesised within a common relative-risk framework.

The primary exposure was daily outdoor activity time. For each study, risk estimates were extracted comparing groups with differing outdoor exposure durations. When available, exposure-response data were collected to characterise the association between increasing outdoor time and myopia occurrence.

### Risk of bias assessment

Risk of bias was evaluated using design-appropriate tools. Two investigators independently assessed each study, and discrepancies were resolved through consensus.

For cross-sectional studies, the AXIS tool was applied [15]. Studies scoring 17–20 (≥ 85%) were classified as low risk, 13–16 (65–84%) as moderate risk, and ≤12 (<65%) as high risk. For cohort studies, the Newcastle-Ottawa Scale was used, with scores of 7–9 indicating low risk, 5–6 moderate risk, and ≤ 4 high risk. For randomised controlled trials, the RoB 2 tool was employed [16]. Trials judged ‘low risk’ across all domains were classified as low risk overall; those with ‘some concerns’ in at least one domain and none rated ‘high risk’ were classified as moderate risk; and any trial with at least one ‘high risk’ domain was classified as high risk overall.

An overall risk-of-bias judgment across included studies was synthesised descriptively. If more than 70% of studies were rated low risk, the overall rating was defined as low; if more than 30% were rated high risk, it was defined as high; otherwise, it was considered moderate.

### Data synthesis

Quantitative analyses were conducted using the meta package in *R*, version 8.2-1 (R Foundation for Statistical Computing, Vienna, Austria). All *P*-values were two-sided, and *P* < 0.05 was considered statistically significant.

Effect sizes were expressed as ORs with 95% CIs. A random-effects model using the restricted maximum likelihood estimator was applied. A random-effects framework was selected a priori because genuine between-study heterogeneity was anticipated due to differences in educational systems, exposure assessment methods, and population characteristics. Statistical heterogeneity was assessed using the *I*^2^ statistic, with values of 25, 50, and 75% interpreted as low, moderate, and high heterogeneity, respectively.

To evaluate potential differences in protective effect according to daily exposure duration, two complementary subgroup meta-analyses were prespecified. Exposure thresholds were defined a priori based on commonly reported daily outdoor duration categories in the primary literature and current school-based policy recommendations in China, allowing clinically interpretable contrasts. Contrast 1 compared three predefined exposure categories: 1–2 hours, 2–3 hours, and > 3 hours per day, using < 1 hour per day as the reference group. Contrast 2 dichotomised studies reporting thresholds around two hours into ≤ 2 hours *vs*. > 2 hours per day to verify the primary exposure cut-point. Studies rated as high risk of bias were excluded from subgroup contrasts in order to minimise bias amplification in pooled estimates. Between-group heterogeneity was assessed using Cochran’s *Q* statistic, followed by pairwise subgroup comparisons. Because these comparisons were prespecified, hypothesis-driven, and limited in number, no adjustment for multiple testing was applied.

Sensitivity analyses were conducted to examine robustness and explore potential sources of heterogeneity. First, stratified analyses were performed according to study design (cross-sectional, cohort, randomised interventional), using separate random-effects models within each stratum. Second, exploratory stratification by geographic latitude was conducted, grouping studies into low (< 30°), mid (30–35°), and high (> 35°) latitude categories. Latitude was used as a proxy for ambient daylight intensity and seasonal variation, which may influence cumulative light exposure independent of reported behavioural duration.

For each stratification, pooled odds ratios were estimated using the same restricted maximum likelihood estimator as the primary analysis. Between-group differences were evaluated using Cochran’s *Q* test, and heterogeneity within strata was quantified using *I*^2^. All sensitivity analyses applied identical inclusion criteria and model specifications as the primary analysis.

### Certainty of evidence

Certainty of evidence was evaluated using the Grading of Recommendations, Assessment, Development, and Evaluation (GRADE) framework [17]. Initial certainty was determined by study design – randomised trials beginning as high certainty and observational studies as low – and subsequently downgraded or upgraded based on predefined domains, including risk of bias, inconsistency, indirectness, imprecision, and potential publication bias.

Publication bias was assessed visually using a contour-enhanced funnel plot. Final certainty ratings were determined through investigator consensus after considering the overall body of evidence.

## RESULTS

### Study selection and characteristics

**Figure 1:**
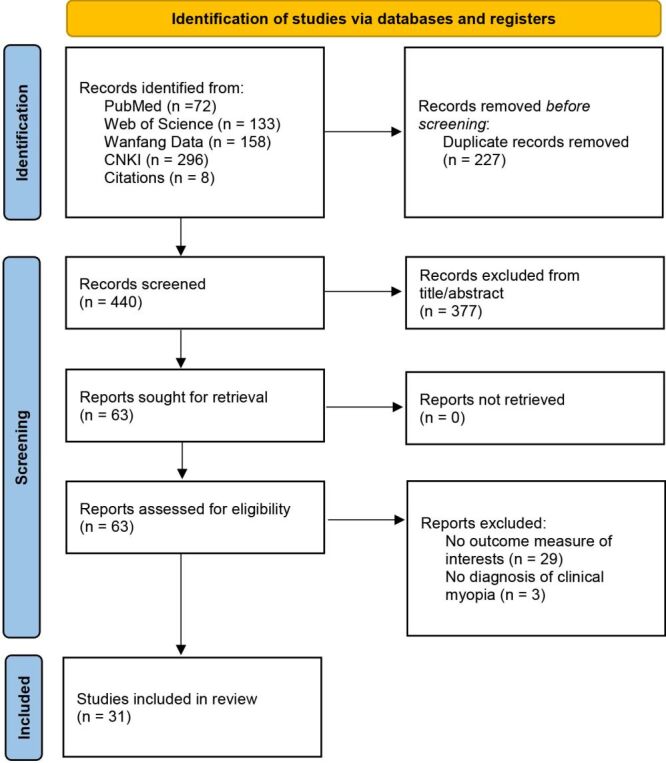
Flowchart of literature search.

Thirty-one studies met the inclusion criteria and were incorporated into the meta-analysis, including 17 English-language [18–34] and 14 Chinese-language [35–48] publications (**Figure 1**).

Detailed characteristics are summarised in **Table 1**. The included studies encompassed diverse designs and populations ranging from preschool to late adolescence, representing 380 215 participants across mainland China. Outdoor exposure was predominantly measured through parent- or self-reported questionnaires, with substantial variability in exposure contrast definitions.

**Table 1:** Characteristics of included studies

Source	Region (Lat.)	Design	N	Age, in years	Myopia prevalence, %	Daytime outdoor exposure	
						**Assessment**	Time (h/d)*
Guo, 2013 [18]	Beijing (40°)	CS	681	5–13	–	Quest.	–
He, 2015 [19]	Guangzhou (23°)	RCT	1579	6.6	34.6	Per class	~ 0.67 *vs. *~* *1.33
Lyu, 2015 [20]	Beijing (40°)	CS	4249	8.9	36.7	Quest.	≦ 1 *vs.* 1–2, >2
Wu, 2015 [21]	Beijing (40°)	Coh.	4292	8.5	51.0	Quest.	–
Guo, 2017 [22]	Beijing (40°)	Coh.	305	10.3	17.3	Quest.	–
Lin, 2017 [23]	Handan (37°)	CS	572	10.6	24.1	Quest.	2.8 *vs.* 3.0
Zhai, 2017 [35]	MC	CS	12 603	12–18	69.6	Quest.	–
Sun, 2018 [24]	Qingdao (36°)	CS	3753	10–15	52.0	Quest.	–
Jiang, 2021 [25]	Wenzhou (28°)	Coh.	438	7	23.3	Quest.	≦ 1.5 *vs.* >2.5
Tang, 2022 [36]	Shanghai (31°)	CS	2036	6–18	54.6	Quest.	<0.5 *vs.* 0.5–1, >1
Xu, 2022 [37]	Guangzhou (23°)	Coh.	1868	6–8	11.4	Quest.	–
Zhong, 2022 [38]	MC	Coh.	3953	6–11	27.5	Quest.	<1* vs*. 1–2, 2–3, ≧ 3
Mu, 2023 [26]	Shenzhen (23°)	Coh.	7597	9–11	51.9	Quest.	1.14 *vs.* 2.68
Zhang, 2023 [27]	Shenyang (42°)	CS	19 882	11.9	72.6	Quest.	<1 *vs.* 1–2, ≧ 2
Zhou, 2023 [28]	Nantong (32°)	CS	2918	15.2	94.0	Quest.	<0.5 *vs.* 0.5–1, 1–2, >2
Dong, 2024 [39]	Xi'an (34°)	CS	5768	12.3	42.5	Quest.	<2 *vs.* ≧ 2
Jiang, 2024 [40]	–	CS	1306	6–12	31.6	Quest.	–
Li, 2024 [29]	Chongqing (30°)	CS	871	12	73.1	Quest.	–
Li, 2024 [29]		CS	871	15	81.8	Quest.	–
Yang, 2024 [30]	Shanghai (31°)	Coh.	2860	8.2	17.5	Device	≦ 1.5 *vs.* 1.5–2.0, >2.0
Yang, 2024 [30]	Shanghai (31°)	Coh.	1552	8.2	17.5	Device	≦ 1.5 *vs.* 1.5–2.0, >2.0
Ye, 2024 [31]	MC	CS	5467	11.2	70.7	Quest.	≦ 1 *vs.* 1–2, 2–3, 3–4, >4
Zhao, 2024 [41]	Jiyuan (35°)	CS	2854	6–18	57.6	Quest.	<2 *vs.* ≧ 2
Gao, 2025 [32]	Chuzhou (32°)	CS	2066	14.6	85.0	Quest.	0.86 *vs.* 1.29
Huo, 2025 [42]	Shandong (37°)	CS	596	7–8	20.6	Quest.	0.3 *vs.* 0.5
Kou, 2025 [43]	MC	CS	9043	10.1	48.3	Quest.	<1 *vs.* >2
Li, 2025 [33]	Tianjin (39°)	Coh.	219 802	8.4	59.3	Quest.	≦ 1 *vs.* 1–2, 2–3, ≧ 3
Li, 2025 [34]	Shantou (23°)	CS	388	4–6	25.7	Quest.	<1 *vs.* 1–2, >2
Tian, 2025 [44]	Shanghai (31°)	CS	8621	12.6	67.5	Quest.	0 *vs.* ≦ 1, 1–2, 2–3, ≧ 3
Wang, 2025 [45]	Shanghai (31°)	CS	28 654	4–18	58.4	Quest.	<2 *vs.* ≧ 2
Wei, 2025 [46]	Liuzhou (24°)	CS	4821	6–18	58.1	Quest.	≦ 2 *vs.* >2
Zhang, 2025 [47]	Nanchang (29°)	CS	15 518	6–18	49.2	Quest.	–
Zheng, 2025 [48]	Chongqing (30°)	CS	2431	6–19	50.1	Quest.	<2 *vs.* 2

Coh. – cohort, CS – cross-sectional, Lat. – latitude, MC – multi-centre, Quest. – questionnaire, RCT – randomised controlled trial

*Outdoor activity time for myopia group *vs.* non-myopia group.

### Overall meta-analysis and risk of bias assessment

**Figure 2:**
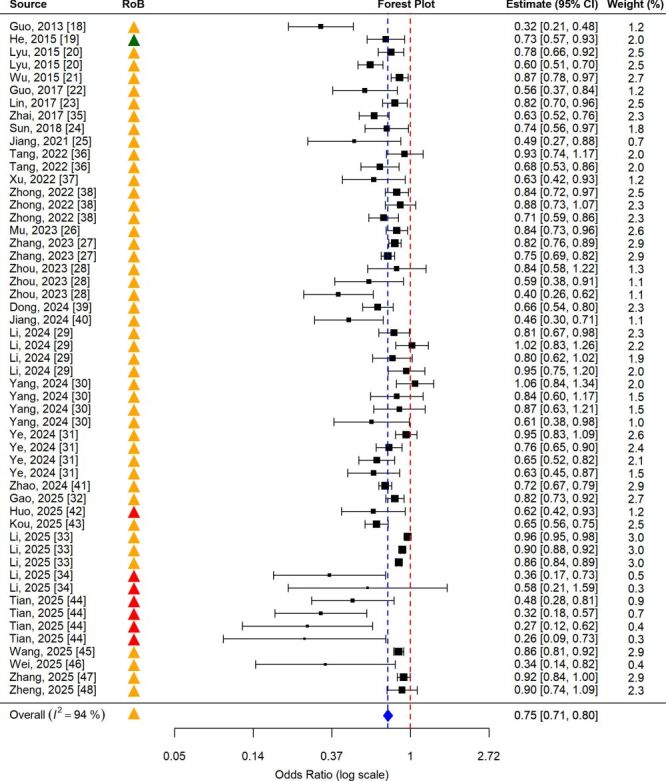
Forest plot of pooled estimates for the association between outdoor activity time and myopia risk, color-coded by risk of bias (RoB). Triangle markers in the ‘RoB’ column denote risk of bias level (green = low, yellow = moderate, red = high). Each square represents an individual study estimate, with the square size proportional to its weight. The blue diamond indicates the pooled overall effect and its 95% confidence interval, the blue dashed vertical line marks the pooled mean estimate, and the red dashed vertical line represents the null reference (odds ratio = 1).

The pooled estimate (**Figure 2**) demonstrated a significant protective association between greater outdoor activity time and lower myopia risk, with an OR of 0.75 (95% CI = 0.71–0.80), indicating that children spending more time outdoors had about a 25% lower risk of developing myopia. However, statistical heterogeneity was high (*I*^2^ = 94%), largely reflecting variation in contrast definitions (*e.g. *< 2 hours *vs*. > 2 hours, < 1 hour *vs*. > 3 hours) and the predominance of cross-sectional designs, which limit temporal inference and confounder control.

The overall risk of bias was rated as moderate. Most studies were methodologically sound but shared common weaknesses, including reliance on self-reported exposure and incomplete adjustment for confounders.

### Sensitivity analysis

Sensitivity analyses stratified by study design and geographic latitude are presented in **Table 2**. Across all stratifications, greater outdoor activity time was consistently associated with a lower risk of myopia, indicating that the primary findings were robust to variation in key study-level characteristics.

**Table 2:** Sensitivity analyses stratified by study design and geographic latitude

Stratification factor	Subgroup	K	Pooled OR (95% CI)	*I*^2^ (%)	Pairwise comparison	*Q*	*P*-value
Study design	CS	22	0.709 (0.655–0.768)	85.0	CS *vs.* Coh.	15.2	<0.0001
	Coh.	8	0.861 (0.814–0.911)	88.3	CS *vs.* RCT	0.0	0.824
	RCT	1	0.730 (0.575–0.927)	0.0	Coh. *vs.* RCT	1.7	0.189
Geographic latitude	Low (< 30°)	6	0.713 (0.575–0.885)	77.5	Low *vs.* Mid	0.2	0.690
	Mid (30–35°)	11	0.749 (0.675–0.831)	79.8	Low *vs.* High	0.2	0.631
	High (> 35°)	9	0.757 (0.674–0.851)	98.6	Mid *vs.* High	0.0	0.885

CI – confidence interval, Coh. – cohort, CS – cross-sectional, K – number of studies included, OR – odds ratio, RCT – randomised controlled trial

Stratification by study design showed that the protective association was observed in cross-sectional, cohort, and randomised interventional studies. Although the magnitude of association differed by design, the direction of effect was consistent across all evidence types. Pairwise subgroup comparisons indicated a significant difference between cross-sectional and cohort estimates, whereas no significant differences were observed between interventional studies and either observational design, supporting the robustness of the association across methodological contexts

Exploratory stratification by geographic latitude likewise demonstrated consistent protective associations across low-, mid-, and high-latitude regions. No statistically significant differences were detected between latitude strata, suggesting that the association between outdoor activity and myopia risk was broadly consistent across diverse ambient light environments. Substantial heterogeneity persisted within strata, indicating that latitude alone does not account for between-study variability.

### GRADE results

**Figure 3:**
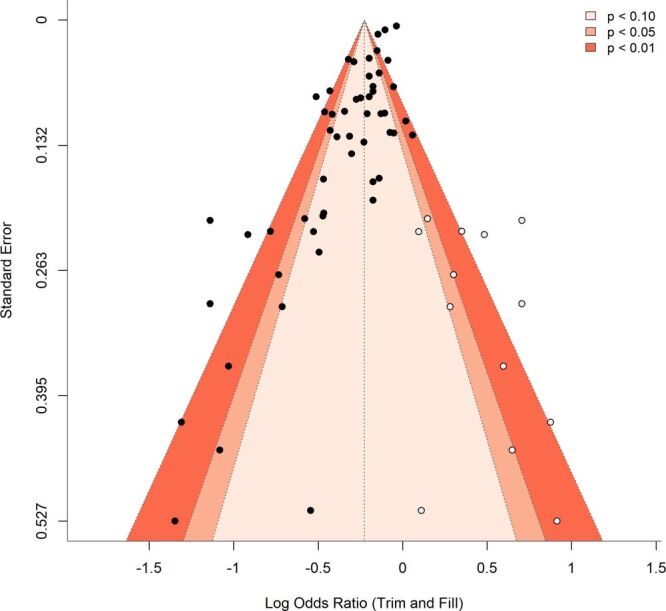
Contour-enhanced funnel plot of the studies included in the meta-analysis, illustrating potential publication bias and results from trim-and-fill adjustment. Each open circle represents an individual study plotted by effect size and standard error. The vertical dashed line marks the pooled overall estimate. The filled circles indicate observed studies, and the open circles represent imputed studies added to restore funnel plot symmetry. Shaded contours correspond to statistical significance regions based on two-sided tests.

Evidence certainty was evaluated using the GRADE framework. As most studies were observational, the initial certainty was low. Risk of bias was not considered serious, since about two-thirds were rated low or moderate risk. Inconsistency was judged serious due to high heterogeneity (*I*^2^ = 94%), although the association remained consistently protective. Indirectness and imprecision were not concerns, as all studies examined relevant populations and the pooled estimate showed a narrow CI (95% CI = 0.71–0.80) excluding the null. The contour-enhanced funnel plot (**Figure 3**) revealed slight asymmetry, suggesting possible small-study effects, but larger studies were symmetrically distributed around the pooled estimate, supporting the robustness of results. Considering all domains, the overall certainty of evidence was rated as moderate, reflecting upgrading for large cumulative sample size and consistency in effect direction.

### Optimal daily exposure time

Exposure-response analyses (**Table 3**) revealed a significant protective association between outdoor activity time and myopia risk. In Contrast 1, children who spent more than three hours [31,33,38] outdoors daily had a pooled OR of 0.74, whereas those with 1–2 hours [19,20,27,28,31–33,38] or 2–3 hours [31,33,38] showed ORs of 0.85 and 0.86, respectively. Although the point estimate for > 3 hours suggested stronger protection, differences among the three exposure strata were not statistically significant, likely reflecting the smaller number of available studies. In Contrast 2, comparing children with >2 hours [20,25–28,30,31,33,38,39,41,43,45,46,48] *vs*. < 2 hours [19,20,27,28,30–33,38] of outdoor activity, the pooled OR was 0.74, confirming that risk reduction becomes substantial once daily outdoor exposure exceeds approximately two hours. Collectively, these results indicate that the protective association appears to plateau once daily exposure approaches approximately two hours, with limited additional benefit observed at higher durations.

**Table 3:** Subgroup analysis of outdoor time and myopia risk

Outdoor exposure	K	N_1_	N_2_	Pooled OR (95% CI)	*I*^2^ (%)	Pairwise comparison	*Q*	*P*-value
Contrast 1*								
*1–2 h*	8	70 576	143 758	0.848 (0.785–0.917)	75.2	1–2 h *vs.* 2–3 h	0.1	0.789
*2–3 h*	3	60 713	25 499	0.863 (0.781–0.954)	51.3	2–3 h *vs.* >3 h	2.6	0.105
*>3 h*	3	60 713	10 587	0.736 (0.625–0.867)	71.9	1–2 h *vs.* >3 h	2.3	0.126
Contrast 2								
*<2 h*†	9	73 442	144 788	0.862 (0.802–0.926)	70.4	<2 h *vs.* >2 h	222.3	<0.0001
*>2 h*‡	15	88 902	75 581	0.744 (0.692–0.801)	90.7	–	–	–

CI – confidence interval, K – number of studies included, N_1_ – total participants in the reference group, N_2_ – total participants in the contrast group, OR – odds ratio

*Reference group: <1 h/d outdoor activity.

†Reference group: <1.5 h/d outdoor activity.

‡Reference group: <2 h/d outdoor activity.

## DISCUSSION

This meta-analysis provides evidence that greater outdoor activity time is associated with a lower risk of myopia among Chinese children and adolescents. Across diverse study designs and settings, increased outdoor exposure was directionally protective, supporting outdoor activity as an important modifiable factor in myopia prevention. At the same time, the findings should be interpreted in the context of substantial heterogeneity and the predominance of observational evidence.

Although most included studies were cross-sectional, sensitivity analyses stratified by study design demonstrated that the protective association was consistently observed across cross-sectional, cohort, and randomised interventional studies. While the magnitude of association differed by design, the direction of effect remained stable, and interventional evidence supported a protective role of outdoor exposure. These findings are consistent with well-designed randomised trials among East Asian children [49,50] demonstrating reduced incident myopia with structured outdoor exposure, and they align with the broader consensus that outdoor activity represents a key modifiable factor in myopia prevention [51,52]. Together, this triangulation across study designs reduces the likelihood that the observed association is attributable solely to cross-sectional bias, although causal inference remains constrained by residual confounding and measurement limitations.

This analysis advances existing knowledge by characterising the exposure-response pattern within the Chinese context, where educational intensity, environmental constraints, and behavioural norms differ considerably from those in other countries. Across studies, protective associations became more consistently apparent once daily outdoor exposure approached approximately two hours, with attenuation of additional benefit at higher levels. This pattern suggests that a pragmatically achievable range of exposure may confer meaningful visual health benefits even within highly competitive academic environments.

The observed exposure-response relationship can be explained through several biological mechanisms. Although direct human evidence is limited, animal studies suggest that bright light regulates retinal dopamine activity, while human research consistently shows that greater sunlight exposure is associated with reduced axial elongation in children [53–55]. Outdoor light also supports circadian regulation by stabilising melatonin-dopamine rhythms that influence ocular growth signalling [56–58], thereby facilitating stable emmetropisation. The apparent plateau in benefit beyond three hours may reflect saturation of light-mediated retinal signalling or a behavioural ceiling beyond which additional outdoor exposure yields limited incremental benefit. This nonlinear, threshold-like pattern is consistent with global evidence summarised by the International Myopia Institute [59], which likewise emphasises moderate outdoor light exposure as a biologically efficient range for slowing ocular growth.

Beyond biological pathways, behavioural mechanisms also contribute substantially to the observed relationship. Increasing outdoor activity naturally reduces time available for near work or screen use-behaviours strongly associated with myopia progression. The approximate 2–3-hour exposure range may reflect a pragmatic equilibrium at which both high-intensity light exposure and reduced near work jointly confer protection. Importantly, this empirically derived range aligns with current Chinese school health policies [60], which recommend a minimum of two hours of daily outdoor activity – including at least one hour of physical education – providing both biological and policy-level justification for their continued implementation.

Two major limitations merit consideration. First, in the included studies, outdoor exposure was predominantly assessed using self-reported questionnaires rather than objective light measurements. Such self-report instruments are subject to recall and social desirability bias and may have introduced exposure misclassification at the primary study level.

Second, substantial heterogeneity persisted even after subgroup and design-stratified analyses. This variability likely reflects multiple, overlapping sources beyond differences in exposure definitions. Included studies varied considerably in age distribution, baseline myopia prevalence, geographic latitude, outcome definitions, and covariate adjustment strategies. In particular, developmental stage may modify susceptibility to outdoor exposure; however, formal age-stratified synthesis was not feasible because age was inconsistently reported and frequently spanned broad and overlapping ranges. Existing evidence suggests that exposure thresholds may differ across grade levels [29,38], potentially reflecting both biological sensitivity during early emmetropisation and escalating academic load in higher grades. Additionally, residual confounding from factors such as near work duration, parental myopia, and socioeconomic status cannot be excluded. Importantly, heterogeneity appeared to influence effect magnitude rather than effect direction, as the protective association was consistently observed across strata.

From a practical standpoint, the present findings support increasing children’s daily outdoor time to around two hours as a feasible and potentially scalable public-health strategy for reducing myopia risk. This target appears achievable within existing educational systems through modest adjustments to school environments, schedules, and behavioural reinforcement, rather than through major structural reform. While such approaches may yield meaningful long-term public-health benefits, formal cost-effectiveness estimates remain limited [61] and context-dependent.

One promising approach involves redesigning school environments to facilitate learning under natural light conditions. Yi et al. [62] demonstrated the feasibility of integrating outdoor or semi-transparent canopies within existing school infrastructure, allowing students to receive high-intensity light exposure during regular academic activities without compromising instructional quality. By embedding outdoor or naturally lit spaces into daily routines, schools may increase cumulative light exposure while maintaining academic engagement.

Adjusting class schedules and recess structures offers another accessible strategy. Chen et al. [63] showed that utilising a continuous 15-minute recess period for class transitions increased total daily outdoor exposure and reduced myopic shift. Encouraging students to spend recesses outdoors can help them approach recommended exposure levels without reducing instructional time, particularly in densely populated urban schools where opportunities for prolonged outdoor sessions are limited [64].

Technology-enabled approaches may further support adherence and monitoring. Digital reminders and wearable devices equipped with ambient-light sensors offer potential tools for tracking cumulative exposure and reinforcing behaviour [54], although additional research is needed to evaluate their long-term effectiveness, equity, and scalability across diverse settings.

Geographic and seasonal considerations should also be incorporated into implementation strategies, as differences in daylight availability and climate may influence feasibility, even though latitude-stratified analyses did not indicate statistically significant effect modification. Nonetheless, schools at high latitudes could prioritise outdoor activity during midday hours, or explore the use of safe, high-intensity artificial lighting that mimics the spectral qualities of daylight to compensate for seasonal deficits.

## CONCLUSIONS

In conclusion, this meta-analysis supports a consistent inverse association between outdoor activity time and myopia risk among Chinese children and adolescents. Across diverse study designs and geographic settings, greater outdoor exposure was associated with lower odds of myopia, although effect sizes varied and substantial heterogeneity persisted.

Promoting regular outdoor activity – on the order of approximately two hours per day – represents a reasonable, evidence-supported public-health target derived from heterogeneous observational and interventional data. This exposure range should be interpreted as an approximate and pragmatic benchmark rather than a rigid or universal optimum. Future longitudinal and experimental studies with standardised exposure assessment and age-specific analyses will be essential to refine exposure thresholds, clarify effect modification, and strengthen causal inference. Coordinated efforts by educators, parents, and policymakers remain critical to embedding outdoor exposure into children’s daily routines through curriculum design, recess scheduling, and school-based health initiatives.

## Additional material


Online Supplementary Document
