# Supplementary material for: Exposure-response association between outdoor activity time and myopia risk in Chinese children and adolescents: a systematic review and meta-analysis
Source: J Glob Health. 2026 Apr 3;16:04122. doi: 10.7189/jogh.16.04122 (PMC13047349; doi:10.7189/jogh.16.04122)
Supplement: Online Supplementary Document [file jogh-16-04122-s001.pdf]

Supplement to: Dai J, Wang J, Bao Y, Zhang Y. Exposure-response association between outdoor activity time and myopia risk in Chinese children and adolescents: a systematic review and meta-analysis. J Glob Health. 2026;16:04122.

Table of Contents

Table/Figure/Method	Title	Page
Text S1	Explanation of authorship change statement	1

Text S1. Explanation of the 'authorship change statement'

Initially, the authors agreed to share corresponding authorship between Jing Wang and Yong-Hong Bao. Upon revision, and with the consent of all authors, Jing Wang and Yang Zhang have been designated as the final corresponding authors to better reflect their contributions and responsibilities in the manuscript.
